# The histone acetyltransferase GCN5 and the transcriptional coactivator ADA2b affect trichome initiation in *Arabidopsis thaliana*

**DOI:** 10.17912/micropub.biology.000176

**Published:** 2019-10-17

**Authors:** Amy T Hark, Elizabeth R McCain1

**Affiliations:** 1 Biology Department, Muhlenberg College, Allentown, PA 18104

**Figure 1:**
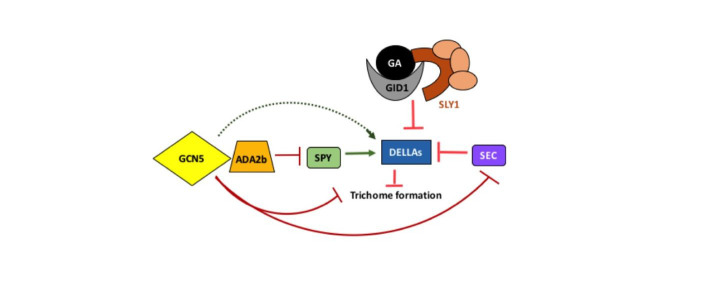
Relationships between players that affect trichome initiation in *Arabidopsis thaliana*. Gibberellin (GA) binds to the receptor GID1 and forms an association with the DELLA repressors, which themselves act to block trichome formation. The GA-GID1-DELLA complex interacts with SLEEPY1 (SLY1). SLY1 is a component of a complex that polyubiqutinylates the DELLAs, resulting in their proteasome-mediated degradation. SPINDLY (SPY) and SECRET AGENT (SEC) have been shown to covalently modify the DELLAs, with opposite effects on activity. Our recent data supports a role for the histone acetyltransferase GCN5 and associated factor ADA2b in inhibiting trichome formation. These chromatin modifiers may work through stimulating expression of one of the DELLA proteins. Our data also indicate that GCN5 and ADA2b block expression of *SEC* and that ADA2b affects *SPY*, suggesting additional input from these epigenetic factors in regulation of the trichome initiation pathway.

## Description

This integrations article considers data from Kotak *et al.* 2019 and Trachtman *et al.* 2019. Our interest is in understanding the place of chromatin modifiers in developmental control pathways, working in concert with or in parallel to established transcriptional factor networks, hormonal signaling, etc.

Previously we have described how the histone acetyltransferase GCN5 and its associated transcriptional coactivator ADA2b affect trichome morphogenesis (Kotak *et al.* 2018). We have now shown that these epigenetic factors also play a role in specification of trichome cell fate, as evidenced by an increase in trichome number and/or density in disruption mutant backgrounds (Kotak *et al.* 2019). Wang *et al.* 2019 also recently showed an increase in trichome density on the first pair of true leaves in several *gcn5* mutant backgrounds. Therefore, GCN5 and ADA2b act to limit trichome initiation (Fig. 1) from a field of epidermal leaf cells, a well-described developmental process involving many genes (Pesch and Hulskamp 2009)

In addition, a number of phytohormones affect trichome development (reviewed in Fambrini and Pugliesi 2019). We focused on pathways connected to gibberellin (GA) signaling, which has been shown to stimulate trichome development in Arabidopsis (Chien and Sussex, 1996). Mutations in two members of the DELLA repressor protein family, RGA and GAI, can rescue the glaborous phenotype of *ga1-3* mutants, indicating a role for these proteins in repressing trichome formation (Dill and Sun, 2001). The DELLAs themselves are negatively regulated by GA in concert with SLY1, which induces degradation via the proteasome pathway (Dill 2004) and by SEC, which has been shown to covalently modify RGA (Zentella *et al.* 2016). SPY also covalently modifies the DELLAs, in a way that promotes their activity (Zentella *et al.* 2017, Fig. 1).

With disruption of *GCN5* or *ADA2b*, GAI expression in rosette leaves is slightly decreased while there is no detectable change in RGA expression (Trachtman *et al.* 2019; Vlachonasios *et al.* 2003). Decreased expression of a DELLA repressor would be consistent with the increased number and density of rosette leaf trichomes observed in *gcn5* and *ada2b* disruption mutant backgrounds (Kotak *et al.*2019, Wang *et al.* 2019, Fig. 1). However, given the modest observed effects on expression of GAI only, it seems unlikely that this change alone fully explains GCN5’s and ADA2b’s roles in trichome initiation. Gan et al. (2007) have reported that GAI also has a role in limiting trichome branching so the effects on GAI may also relate to trichome morphogenesis.

We also show that disruption of *GCN5* or *ADA2b* leads to an increased expression of *SEC* (Trachtman *et al.* 2019, Fig. 1). This effect is consistent with increased trichome number in *gcn5* and *ada2b* disruption mutants. Our data suggest a role for ADA2b in limiting expression of *SPY*, which would be expected to *decrease* trichome number. This transcriptional effect on *SPY* may relate to the decrease in absolute number of trichomes observed only in the second true leaf in an *ada2b-1* mutant background (Kotak *et al.* 2019). This finding could be further explored by conducting expression analysis in the first and second true leaves separately. It should also be noted that since qRT-PCR experiments do not directly measure functional protein levels, there could be translational or post-translational effects to consider.

While data from *gcn5-1* and *gcn5-6* is generally consistent, somewhat different effects (as seen with SLY1; Trachtman *et al.* 2019) may expected due to the nature of these lesions in the *GCN5* locus and/or the genetic backgrounds (Ws vs Col ecotypes, respectively). It is also important to note that we examined mature rosette leaves, as it is technically difficult to isolate trichomes for this analysis, especially from the mutant plants. This may result in variability seen in some experiments (e.g. SEC in *gcn5-1*; Trachtman *et al.* 2019).

This work places epigenetic players in a developmental pathway alongside other transcriptional regulators. To uncover more details about how these factors act and interact, chromatin immunoprecipitation could be used to assess changes in histone acetylation state or potentially GCN5 and ADA2b binding at the GAI, SEC, and SPY promoter, to determine if these loci are direct targets of these chromatin modifiers.
